# Hypokalemic Paralysis Secondary to Renal Tubular Acidosis Revealing Underlying Sjogren's Syndrome

**DOI:** 10.7759/cureus.3128

**Published:** 2018-08-10

**Authors:** Amir Shahbaz, Muhammad Faizan Shahid, Hafiz M. Kashif Saleem, Zohra R Malik, Issac Sachmechi

**Affiliations:** 1 Internal Medicine, Icahn School of Medicine at Mount Sinai/Queens Hospital Center, New York, USA; 2 Internal Medicine, Jinnah Hospital/Allama Iqbal Medical College, Lahore, PAK; 3 Internal Medicine, Allama Iqbal Medical College, Lahore, PAK; 4 Internal Medicine, Icahn School of Medicine at Mount Sinai/Queens Hospital Center, New York City, USA

**Keywords:** sjogren's syndrome, renal tubular acidosis, hypokalemia

## Abstract

There is a well-established association of Sjogren's syndrome with renal tubular acidosis (RTA). Rarely there is a retrospective diagnosis where the patient presents with RTA and the workup reveals Sjogren's syndrome. Our case report is about a patient who presented with generalized weakness and hypokalemia, which upon further workup turned out to be RTA. Various tests were performed to find out the cause of RTA. A favorable profile for the anti-nuclear antibody, anti-Ro/SSA, and anti-La/SSB was consistent with Sjogren's syndrome. Treatment with corticosteroid improved hypokalemia. The patient did not have typical glandular symptoms of Sjogren's syndrome, and follow-up is needed to see whether the patient develops symptoms in the future and to prevent any possible complication.

## Introduction

Sjogren's syndrome is a systemic autoimmune disease characterized by cell-mediated autoimmunity against exocrine glands. Involvement of the renal system in Sjogren's syndrome is one of the extra-glandular manifestations. Although distal renal tubular acidosis (dRTA) is common in Sjogren's syndrome, it is usually asymptomatic [1]. The clinical spectrum of renal tubular acidosis may be silent to life-threatening [2]. The most common electrolyte abnormality in distal renal tubular acidosis is hypokalemia, occurring in approximately 28–53% of patients [3]. Hypokalemia may occur earlier than typical glandular symptoms and reveal a previously undiagnosed Sjogren's syndrome.

## Case presentation

A 28-year-old female presented with complaints of vomiting for three days. For the last one day, the patient was bedridden secondary to extreme fatigue and weakness. She denied fever, vaginal discharge, or diarrhea. Her blood pressure was 100/60 mmHg, respiratory rate 22/min, heart rate 56/min, and she was conscious and alert. An examination of her nervous system revealed that there was proximal muscle weakness and that her reflexes were flaccid. There was no evident muscle tenderness or sensory deficit. Her blood work was normal except for low serum bicarbonate (7.0 mmol/L) and serum potassium (1.5 mmol/L). A spot urine sample showed a pH of 6.5 and a positive anion gap (urinary sodium 5.1 mmol/L, potassium 34 mmol/L, and chloride < 5 mmol/L). On arterial blood gas analysis, the pH was 7.04 (7.35-7.45), _p_CO_2_ 6.1 kPa (4.5-6.1), _P_O_2_ 12.8 kPa (12.0-15.0), bicarbonate 12.0 mmol/L (22.0-26.0), and oxygen saturation 92.6%. All of these findings were compatible with the diagnosis of dRTA.

While receiving therapy with potassium replacement, the patient developed increasing shortness of breath from respiratory muscle weakness. Repeat biochemical analyses showed serum potassium of 1.8 mmol/L. She was transferred to the intensive care unit and intubated for ventilation. Further tests were arranged to establish the cause of dRTA. On serum protein electrophoresis, we did not find paraproteins. Ultrasound of the abdomen was normal, excluding medullary sponge kidney, nephrocalcinosis, and obstructive uropathy as potential causes of dRTA. Her autoantibody profile showed negative anti-mitochondrial, anti-smooth muscle, and anti-double-stranded DNA antibodies. However, anti-nuclear antibody, anti-Ro/SSA, and anti La/SSB were positive. This autoantibody pattern was consistent with Sjogren's syndrome. There was no history of dry eyes and dry mouth. We did not perform a Schirmer test or salivary gland biopsy. Therapy with intravenous corticosteroids was started while the potassium replacement continued. Her condition improved and her hypokalemia resolved. The patient was extubated and discharged after one week with the advice of follow-up.

## Discussion

Deficient serum potassium may rarely lead to sudden life-threatening paralysis and respiratory failure and can reveal a previously undiagnosed disease process. When a patient presents with severe hypokalemia, evaluation of the acid-base status helps in narrowing the differential diagnosis. Measurement of serum bicarbonate and chloride establish the diagnosis of hypokalemic hyperchloremic metabolic acidosis [4]. A random urinary pH of 6.5, while the patient was acidotic, confirmed dRTA as the underlying mechanism [5]. dRTA is characterized by an inability of the kidneys to excrete hydrogen ions in the urine in a setting of metabolic acidosis resulting in inappropriately normal or alkaline urine. A normal anion-gap metabolic acidosis, positive urine anion gap, and hypokalemia diagnose the condition [6]. Evidence of salivary and lacrimal gland involvement, marked lymphocytic infiltration on salivary gland biopsy, and the presence of Ro/SSA or La/SSB autoantibodies establish the diagnosis of Sjogren's syndrome [7]. Our patient had an antibody profile consistent with Sjogren's syndrome despite the absence of the sicca symptoms. It is possible that dRTA as an extra-glandular manifestation of Sjogren's syndrome with positive antibody profile precedes the glandular symptoms of dry eye and mouth several months or even years [5,8,9].

Sjogren's syndrome is a systemic autoimmune disease characterized by cell-mediated autoimmunity against exocrine glands. Extraglandular manifestations of the disease arise from a similar pathogenetic mechanism affecting the kidneys, liver, lungs, pancreas, and the nervous system [10]. Renal involvement in Sjogren's syndrome is rare, affecting <10% of patients [11]. dRTA in Sjogren's syndrome is usually asymptomatic and goes undetected in most cases [1]. Hypokalemic paralysis in association with Sjogren's syndrome is rare and only occasionally is it the sole primary manifestation [6]. In Sjogren's syndrome, severe symptomatic hypokalemia leading to a respiratory arrest has been reported in only a few cases [5,6].

The pathogenesis of dRTA in Sjogren's syndrome is autoimmune tubulointerstitial nephropathy [2]. dRTA is treated with potassium administration and alkali therapy with bicarbonate [12]. Corticosteroids and immunosuppressants have been reported to slow progression of renal damage in Sjogren's syndrome, but the efficacy of the treatment was demonstrated only in patients with rapidly progressing renal damage or insufficiency [13].

## Conclusions

The diagnosis of Sjogren's syndrome is made by either clinical manifestations or biochemical tests or histological diagnosis. Autoimmune investigations for Sjogren's syndrome should be considered in any patient presenting with hypokalemic paralysis from dRTA, even in the absence of the sicca symptoms.

## References

[1] Lim AK, Choi MJ (2013). Distal renal tubular acidosis associated with Sjogren syndrome. Intern Med J.

[2] Pessler F, Emery H, Dai L, Wu YM, Monash B, Cron RQ, Pradhan M (2006). The spectrum of renal tubular acidosis in paediatric Sjögren syndrome. Rheumatology (Oxford).

[3] Cherif E, Ben Hassine L, Kechaou I, Khalfallah N (2013). Hypokalemic rhabdomyolysis: an unusual presentation of Sjogren's syndrome. BMJ Case Rep.

[4] Kardalas E, Paschou SA, Anagnostis P, Muscogiuri G, Siasos G, Vryonidou A (2018). Hypokalemia: a clinical update. Endocr Connect.

[5] Al-Jubouri MA, Jones S, Macmillan R, Harris C, Griffiths RD (1999). Hypokalaemic paralysis revealing Sjögren syndrome in an elderly man. J Clin Pathol.

[6] Vaidya G, Ganeshpure S (2012). Sjogren's syndrome with distal renal tubular acidosis presenting as hypokalaemic paralysis. BMJ Case Rep.

[7] Mavragani CP, Moutsopoulos HM (2014). Sjögren syndrome. CMAJ.

[8] Theander E, Jonsson R, Sjöström B, Brokstad K, Olsson P, Henriksson G (2015). Prediction of Sjögren's syndrome years before diagnosis and identification of patients with early onset and severe disease course by autoantibody profiling. Arthritis Rheumatol.

[9] Jonsson R, Theander E, Sjöström B, Brokstad K, Henriksson G (2013). Autoantibodies present before symptom onset in primary Sjögren syndrome. JAMA.

[10] Leone MC, Alunno A, Cafaro G (2017). The clinical spectrum of primary Sjögren's syndrome: beyond exocrine glands. Reumatismo.

[11] François H, Mariette X (2016). Renal involvement in primary Sjögren syndrome. Nat Rev Nephrol.

[12] (2018). Treatment of distal (type 1) and proximal (type 2) renal tubular acidosis. https://www.uptodate.com/contents/treatment-of-distal-type-1-and-proximal-type-2-renal-tubular-acidosis?topicRef=2291&source=see_link.

[13] Maripuri S, Grande JP, Osborn TG (2009). Renal involvement in primary Sjögren's syndrome: a clinicopathologic study. Clin J Am Soc Nephrol.

